# Sensitization of primary cultures from rat dorsal root ganglia with lipopolysaccharide (LPS) requires a robust inflammatory response

**DOI:** 10.1007/s00011-021-01534-2

**Published:** 2021-12-23

**Authors:** Franz Nürnberger, Stephan Leisengang, Daniela Ott, Jolanta Murgott, Rüdiger Gerstberger, Christoph Rummel, Joachim Roth

**Affiliations:** Department of Veterinary-Physiology and -Biochemistry, Justus Liebig University Giessen, Frankfurter Strasse 100, 35392 Giessen, Germany

**Keywords:** LPS sensitization, Dorsal root ganglia, Mixed neuro-glial cultures, Inflammation, Cytokines, Capsaicin, Ca^2+^-imaging

## Abstract

**Objective:**

We investigated whether it is possible to induce a state of “LPS-sensitization” in neurons of primary cultures from rat dorsal root ganglia by pre-treatment with ultra-low doses of LPS.

**Methods:**

DRG primary cultures were pre-treated with low to ultra-low doses of LPS (0.001–0.1 µg/ml) for 18 h, followed by a short-term stimulation with a higher LPS-dose (10 µg/ml for 2 h). TNF-α in the supernatants was measured as a sensitive read out. Using the fura-2 340/380 nm ratio imaging technique, we further investigated the capsaicin-evoked Ca^2+^-signals in neurons from DRG, which were pre-treated with a wide range of LPS-doses.

**Results:**

Release of TNF-α evoked by stimulation with 10 µg/ml LPS into the supernatant was not significantly modified by pre-exposure to low to ultra-low LPS-doses. Capsaicin-evoked Ca^2+^-signals were significantly enhanced by pre-treatment with LPS doses being above a certain threshold.

**Conclusion:**

Ultra-low doses of LPS, which per se do not evoke a detectable inflammatory response, are not sufficient to sensitize neurons (Ca^2+^-responses) and glial elements (TNF-α-responses) of the primary afferent somatosensory system.

**Supplementary Information:**

The online version contains supplementary material available at 10.1007/s00011-021-01534-2.

## Introduction

Pre-exposure of macrophages to LPS causes either tolerance, meaning that the responses to a second LPS-challenge are strongly attenuated, or priming, an elevated response to a second hit of LPS [1]. Cells or animals become LPS-tolerant when a first challenge with LPS causes a robust inflammatory response. Priming or sensitization is induced by ultra-low LPS-doses, that per se will not evoke substantial formation and release of pro-inflammatory cytokines. A state of LPS-tolerance can also be evoked in structures of the peripheral or central nervous system [2]. Whether or not LPS-sensitization will occur in a given neuroglial structure, especially in dorsal root ganglia (DRG), has not yet been investigated. The central goals of this study can, therefore, be summarized as follows: we first tried to determine ultra-low LPS-doses for a long-term stimulation of DRG cultures for 18 h, which per se did not cause elevations of TNF-α in the supernatants but an enhanced production of this cytokine by a second hit with a high LPS-dose (“sensitization”). We further tested the effects of the presence of ultra-low, moderate and high LPS-doses on the capsaicin-evoked responses of neurons from rat DRG.

## Materials and methods

See supplementary material.

## Results

**Fig. 1:**
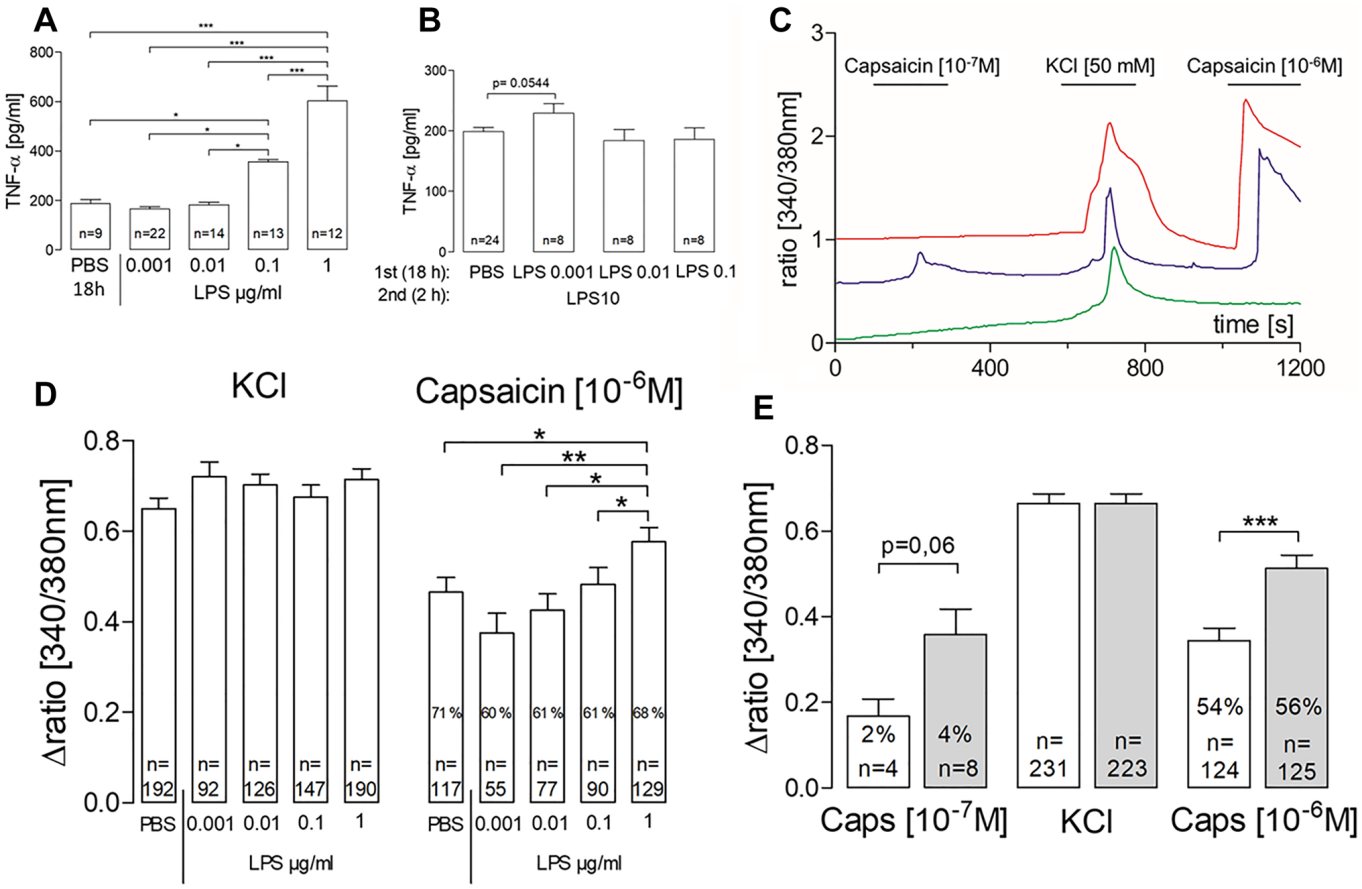
**A**, **B** TNF-α release after incubation with different doses of LPS and PBS: DRG primary cultures were incubated with different doses of LPS (0.001, 0.01, 0.1, 1 µg/ml) for 18 h or PBS as control (**A**) for subsequent measurement of TNF-α in the supernatants. In a second series of experiments (**B**), DRG primary cultures were pre incubated with different doses (0.001, 0.01, 0.1 µg/ml) for 18 h as well as PBS as control followed by a second stimulation with a high LPS dose (10 µg/ml) for 2 h. Pre-incubation with 0.001 μg/ml followed by a stimulation with a high LPS dose showed a tendency of a higher TNF-α release into supernatants compared to control. Each column represents the mean ± SEM of *n* samples and four different experiments. One-way ANOVA followed by a Newman–Keuls multiple comparison test was performed for statistical analysis. The graphical depiction of the *p* values were illustrated as follows: ***p < 0.001; **p < 0.01; *p < 0.05; **D** DRG primary cells were stimulated with different doses (0.001, 0.01, 0.1, 1 μg/ml) of LPS for 18 h and PBS as control. KCl served as vitality control for DRG neurons. ∆ratio [340/380 nm] represents the mean ± SEM increase of intracellular calcium [Ca^2+^]_i_ of *n* cells of six different preparations. Percentages represent the numbers of responsive cells to a distinct stimulus, e.g., capsaicin compared to all vital neurons (KCl). Statistical analysis of KCl or capsaicin responses was performed using a one-way ANOVA followed by a Newman–Keuls multiple comparison test. *p* values were represented as follows: ***p < 0.001; **p < 0.01; *p < 0.05; **C, E** Prior to the experiments DRG primary cultures were incubated with LPS (1 μg/ml for 18 h) or PBS (18 h). After incubation different stimuli (capsaicin [10^−7^ M], KCl and capsaicin [10^–6^ M]) were applied to investigate neuronal responses. **C** Examples of tracings recorded from three DRG neurons: one neuron responding to KCl [50 mM] only (green), one responding to KCl and capsaicin [10^−6^ M] (red) and one responding to all three stimuli KCl, capsaicin [10^−6^ M and 10^−7^ M] (blue). **E** Depicts ∆ratio [340/380 nm] fluorescence values as a measurement of [Ca^2+^]. Columns represent the mean ± SEM increase of intracellular calcium [Ca^2+^]_*i*_ of *n* cells from 4 different preparations. Statistical analysis between the PBS and LPS group was performed using an unpaired *t* test. ***p < 0.001 (color figure online)

Cultivation of DRG primary cultures in presence of various doses of LPS was accompanied by a dose dependent rise of TNF-α in the supernatants (Fig. 1A). The lowest LPS-doses used in this experiment (0.01 and 0.001 µg/ml) did not evoke a significant increase of TNF-α in the supernatants. To test whether primary DRG cultures were sensitized to a subsequent hit with a high LPS dose, cultures were stimulated with 10 µg/ml LPS for 2 h after pre-exposure with 0.1, 0.01 or 0.001 µg/ml LPS for 18 h (Fig. 1B). The slight LPS-induced (10 µg/ml) increase of TNF-α in supernatants of cells pre-treated with 0.001 µg/ml LPS was not significant.

DRG primary cultures were incubated in presence of PBS or LPS at various doses (0.001, 0.01, 0.1 or 1 µg/ml) for 18 h. Thereafter, the strength of stimulus-induced Ca^2+^-signals of DRG-neurons was evaluated [3]. Capsaicin, at a dose of 10^–6^ M, evoked pronounced Ca^2+^-responses in 60–70% of neurons from all groups (Fig. 1D).

The responses of neurons to the depolarizing KCl-solution (vitality-test) was similar in all groups investigated. A significant enhancement of the strength of capsaicin-induced Ca^2+^-signals was exclusively determined in the group, which was pre-treated with the highest LPS-dose (1 µg/ml, Fig. 1D), the same dose, which evoked a profound increase of TNF-α in the supernatants (Fig. 1A). A sensitization of DRG neurons to a nociceptive stimulus (capsaicin) [4] thus was not achieved by pre-treatment with very low LPS-dose, which per se did not evoke an increase of TNF-α production.

We finally tested whether DRG neurons might also show enhanced responses to the threshold-dose of capsaicin. We determined that not a single DRG neuron showed Ca^2+^-responses to capsaicin at doses 10^–9^ and 10^–8^ M. Stimulation with 10^–7^ M capsaicin evoked Ca^2+^-signals just in single neurons. This dose was, therefore, defined as the threshold dose (Fig. 1C).

Just 4 out of 231 DRG neurons (about 2%) responded to 10^–7^ M capsaicin. In cultures pre-treated with 1 µg/ml LPS 8 out of 223 neurons (about 4%) were responsive to the capsaicin threshold dose (Fig. 1D). Vitality of neurons (responses to KCl) was identical in both groups. Again, the responses of DRG neurons to the effective capsaicin-dose of 10^–6^ M was significantly higher in neurons pre-exposed to 1 µg/ml LPS.

## Discussion

Priming of macrophages with a sub-threshold dose of LPS resulted in enhanced release of TNF-α to a second stimulation with a higher LPS-dose [5]. This effect seems to be the basic mechanism for the phenomenon of LPS-sensitization. We aimed to define experimental conditions, which should mimic such an effect in mixed neuro-glial primary cultures from rat DRG. With regard to the formation and release of TNF-α, the outcome was not as clear as we expected. When pre-treated with an ultra-low LPS-dose, an enhanced TNF-α response to a subsequent stimulation with a high LPS-dose was hardly detectable. Still, there is evidence for a sensitization of capsaicin-responsive (nociceptive) sensory neurons from thoracic DRG with LPS resulting in hypersecretion in the upper airways [6]. Therefore, we tested the effects of cultivation of DRG primary cultures in presence of various doses of LPS on the strength of capsaicin-evoked Ca^2+^-signals. To evoke enhanced capsaicin responses, the presence of an amount of LPS in the culture medium is required, which induces a robust release of TNF-α into the supernatant. Sub-threshold doses of LPS failed to induce such an effect. This means that a given inflammatory insult has to reach a certain threshold to cause a sensitization of peripheral nociceptors finally resulting in the manifestation of inflammatory pain. Our observation that ultra-low doses of LPS failed to evoke sensitization of DRG nociceptive neurons (capsaicin-responses) and the mixed neuroglial culture (formation of TNF-α) can possibly be explained by the fact that just 2% of all cells of DRG cultures are macrophages [2]. Cells from the macrophage–monocyte-lineage seem to be critical for sensitization to LPS, also in structures of the nervous system [7, 8]. Future studies should, therefore, employ central nervous structures including a higher percentage of cells from the macrophage–monocyte-lineage to investigate the phenomenon of LPS-sensitization in mixed neuroglial tissue.

## Supplementary Information

Below is the link to the electronic supplementary material.

Supplementary file1 (DOCX 28 KB)
