# Supplementary material for: Sensitization of primary cultures from rat dorsal root ganglia with lipopolysaccharide (LPS) requires a robust inflammatory response
Source: Inflamm Res. 2021 Dec 23;71(2):187–90. doi: 10.1007/s00011-021-01534-2 (PMC8800878; doi:10.1007/s00011-021-01534-2)
Supplement: Supplementary file 1 — Supplementary file1 (DOCX 28 KB) [file 11_2021_1534_MOESM1_ESM.docx]

# Materials and methods

## Animals

For preparation of primary cultures of rat DRG we used 4-6 weeks old Wistar rats from an in-house breeding. Parent animals originated from Charles River WIGA (Sulzfeld, Germany). The experimental setup, breeding and animal care was performed compliant with the German Law on Animal Welfare, authorized by the Justus-Liebig-University of Giessen (approval number GI 577_M) and registered to the regional authority of Hessia. Ambient temperature was kept at 22 ± 1 °C and a relative humidity of 50 ± 5 % was configured. Animals had ad libitum access to water and standard laboratory chow. The artificial light was turned on from 7:00 AM to 7:00 PM.

## Primary culture of rat DRG

DRG primary cultures were prepared as previously described [Leisengang et al. Primary cultures from rat dorsal root ganglia: responses of neurons and glial cells to inflammatory or somatosensory stimuli. Neuroscience. 2018; 394: 1–13]. Under CO_2_ anesthesia rats were killed by cervical dislocation. Redundant skin and muscles and dorsal parts of the vertebral column were removed. 15-20 DRG were sliced out and put into Petri dishes with cold, oxygenated GBSS (Gey’s Balanced Salt Solution; Sigma-Aldrich Chemie GmbH, Taufkirchen, Germany) supplemented with 0.5 % D-Glucose (Sigma-Aldrich Chemie GmbH). Expendable spinal nerves and meninges were removed and the isolated DRG were transferred into HBSS (Hanks Balanced Salt Solution, without Ca^2+^ and Mg^2+^; Biochrom GmbH, Berlin, Germany) supplemented with 20 mM HEPES (Sigma-Aldrich Chemie GmbH) at pH 7.4. DRG were then enzymatically digested using 5 mg/ml dispase II (Sigma-Aldrich Chemie GmbH) and 2.5 mg/ml collagenase (CLS II; Biochrom GmbH) dissolved in 2 ml oxygenated HBSS for 1 h at 37 °C. After enzymatical digestion and mechanical dissociation the cells were washed using HBSS containing 1 mM EDTA (Sigma-Aldrich Chemie GmbH) to stop the enzymes activity. After two washing steps using Neurobasal A medium supplemented with 2 % B27, penicillin (100 U/ml) / streptomycin (0.1 mg/ml) and 2 mM L-glutamine (Life Technologies GmbH, Darmstadt, Germany) DRG were resuspended, cultured with a cell number of 75,000 cells/ml and plated onto poly-L-lysine (0.1 mg/ml; Biochrom GmbH) coated glass coverslips (Menzel, Braunschweig, Germany). After 4 h of cultivation in a humidified atmosphere of 5 % CO_2_ and 95 % air at 37 °C the cells had attached to the glass coverslips. After another washing step, cells were used for experiments.

## Ca^2+^-imaging experiments

After preparation of DRG primary cultures, the cells were incubated with Neurobasal A medium supplemented with 2 % B27, penicillin (100 U/ml) / streptomycin (0.1 mg/ml) and 2 mM L-glutamine (Life Technologies GmbH, Darmstadt, Germany) in presence of distinct LPS-doses or PBS for 18 h. After incubation the cells were loaded with 2 µM fura-2-AM (Life Technologies GmbH) for 40 minutes at a temperature of 37 °C in a humidified atmosphere of 5 % CO_2_/ 95 % air. Afterwards the glass coverslips were implemented in specially constructed Teflon© chambers under an inverted microscope (IMT-2, Olympus GmbH) and washed with Ca^2+^-imaging buffer composed of 5 mM HEPES, 130 mM NaCl, 5 mM KCl, 1.0 mM MgCl_2_, 1.25 mM CaCl_2_, and 10 mM D-glucose (all: Sigma-Aldrich Chemie GmbH) at pH 7.4. The following superfusion with Ca^2+^-imaging buffer was kept constantly at a temperature of 37 °C and a superfusion rate of 2.0 ml/min. The fluorescence measurements were performed using a filter wheel-based excitation system and computed by MetaFluor 7.7.8.0. software (Visitron GmbH, Puchheim, Germany). The respective cells were marked as regions of interest and emitted fluorescence (>515 nm) was detected after altering excitations (340 and 380 nm) every five seconds using a Spot Pursuit digital CCD-camera (Model 23.0, Visitron GmbH). As final readout the 340/380 nm ratios were computed and analyzed.

The TRPV1 agonist capsaicin (10^-9^, 10^-8^, 10^-7^ and 10^-6^ M, 180 s) diluted in Ca^2+^-imaging buffer from a stock solution (10 mM in 0.1 % DMSO) and 50 mM KCl (180 s, vitality test) were used as stimuli for DRG neurons.

## Measurement of TNF-α

Measurement of TNF-α in supernatants from DRG primary cultures is in our hands the most sensitive readout to determine the effects of inflammatory stimulation with LPS [Leisengang et al. Primary culture of the rat spinal dorsal horn: a tool to investigate the effects of inflammatory stimulation on the afferent somatosensory system. Plügers Archiv. 2020;472:1769–82]. For the detection of TNF-α we employed a highly sensitive bioassay using the mouse fibrosarcoma cell line WEHI 164 subclone 13. The underlying principle of the TNF-α bioassay is the cytotoxic effect of TNF-α on this cell line. 50,000 actinomycin D-treated WEHI cells were incubated with serial dilutions of samples or different concentrations of an international standard (murine TNF-α code 88/532, National Institute for Biological Standards and Control, South Mimms, UK) for 24 h in a 96 well plate. After incubation the number of surviving cells was measured using the dimethylthiazol-diphenyl tetrazolium bromide (MTT) colorimetric assay. The detection limit for TNF-α in this bioassay was 6 pg/ml.

## Experimental protocols

Four hours after cultivation cells were stimulated with different doses of LPS (Sigma Aldrich Chemie GmbH) for 18 h, whereas a control group was treated with phosphate buffered saline (PBS, Capricorn Scientific GmbH, Ebsdorfer Grund, Germany). In a first series of experiments DRG cells were exposed to 0.001, 0.01, 0.1, 1 µg/ml LPS or PBS for 18 h and TNF-α release into the supernatants was investigated to identify sub-threshold LPS doses. DRG primary cultures were further stimulated with 0.001, 0.01, 0.1 µg/ml LPS or PBS for 18 h followed by a second stimulation with a high LPS dose (10 µg/ml for 2 h) to identify and characterize a state of LPS-sensitization. This high LPS-dose for the subsequent short-term stimulation was chosen due to previous studies where we detected suitable doses for short-term LPS stimulation. Even higher doses (100 µg/ml) were tested in DRG primary cultures, as well as in primary cultures of the superficial dorsal horn that resulted in an increased cytokine release into the supernatant, but did not impair vitality of our primary cell cultures [Leisengang et al. Primary cultures from rat dorsal root ganglia: responses of neurons and glial cells to inflammatory or somatosensory stimuli. Neuroscience. 2018; 394: 1–13] and [Leisengang et al. Primary culture of the rat spinal dorsal horn: a tool to investigate the effects of inflammatory stimulation on the afferent somatosensory system. Plügers Archiv. 2020; 472:1769–82]. Pre-incubation with 1 µg/ml LPS for 18 h induces a state of LPS-tolerance for a subsequent stimulation with the high LPS dose [Nürnberger et al. Manifestation of lipopolysaccharide-induced tolerance in neuro-glial primary cultures of the rat afferent somatosensory system. Inflamm Res. 2021: 70 (4): 429-444]. Therefore, this dose was not used in this experiment.

In a pilot study for the second series of experiments, we determined threshold doses of capsaicin for the activation of the TRPV1 channel (transient receptor potential), an important pain sensor [Garami et al. Contributions of different modes of TRPV1 activation to TRPV1 antagonist-induced hyperthermia. J Neurosci. 2010; 30: 1435-1440]. Capsaicin doses of 10^-9^, 10^-8^, 10^-7^ and 10^-6^ M were tested. Neuronal responses to KCl (50 mM) and capsaicin (10^-6^M) were investigated in Ca^2+^-imaging experiments after 18 h of pre-incubation with either LPS (0.001, 0.01, 0.1, 1 µg/ml) or PBS. Finally, we stimulated DRG primary cultures with either 1 µg/ml LPS or PBS for 18 h and investigated neuronal responses using the following Ca^2+^-imaging stimulation protocol: capsaicin at the determined threshold dose of 10^-7^M followed by KCl (50 mM) and the effective dose of capsaicin (10^-6^M).

## Evaluation and statistics

Concentration of TNF-α in supernatants and the responses (Δratio [340/380 nm]) in Ca^2+^-imaging experiments were presented as mean ± standard error of the mean (SEM). In Ca^2+^-imaging experiments all cells were included, that showed an increase larger than 0.05 from baseline to a distinct stimulus. Percentages within the bars represent the number of responsive cells compared to all KCl-responsive cells. One-way analysis of variance (ANOVA) followed by a Newman-Keuls post hoc test was used for statistical analysis. In the final Ca^2+^-imaging experiment (Figure 3) an unpaired t-test was used to compare PBS- and LPS-treated groups. Calculations were performed using the software package GraphPad Prism (GraphPad Software Inc., LaJolla, CA, USA). The graphical depiction of the *p* values within every experiment is displayed as follows: ***=p<0.001; **p<0.01; *=p<0.05.
